# Defining the binding interface of Amyloid Precursor Protein (APP) and Contactin3 (CNTN3) by site-directed mutagenesis

**DOI:** 10.1371/journal.pone.0219384

**Published:** 2019-07-18

**Authors:** Xi Peng, John Williams, Philip M. Smallwood, Jeremy Nathans

**Affiliations:** 1 Department of Molecular Biology and Genetics, Johns Hopkins University School of Medicine, Baltimore, Maryland, United States of America; 2 Howard Hughes Medical Institute, Johns Hopkins University School of Medicine, Baltimore, Maryland, United States of America; 3 Department of Neuroscience, Johns Hopkins University School of Medicine, Baltimore, Maryland, United States of America; 4 Department of Ophthalmology, Johns Hopkins University School of Medicine, Baltimore, Maryland, United States of America; Torrey Pines Institute for Molecular Studies, UNITED STATES

## Abstract

The Amyloid Precursor Protein (APP) and Contactin (CNTN) families of cell-surface proteins have been intensively studied in the context of neural development and neuropsychiatric diseases. Earlier studies demonstrated both genetic and biochemical interactions between the extracellular domains of APP and CNTN3, but their precise binding interfaces were not defined. In the present study, we have used binding assays between APP-alkaline phosphatase (AP) fusion proteins and CNTN-Fc fusion proteins, together with alanine substitution mutagenesis, to show that: (i) the second Fibronectin domain (Fn(2)) in CNTN3 mediates APP binding; (ii) the copper binding domain (CuBD) in APP mediates CNTN3 binding; and (iii) the most important amino acids for APP-CNTN3 binding reside on one face of CNTN3-Fn(2) and on one face of APP-CuBD. These experiments define the regions of direct contact that mediate the binding interaction between APP and CNTN3.

## Introduction

**Fig 1:**
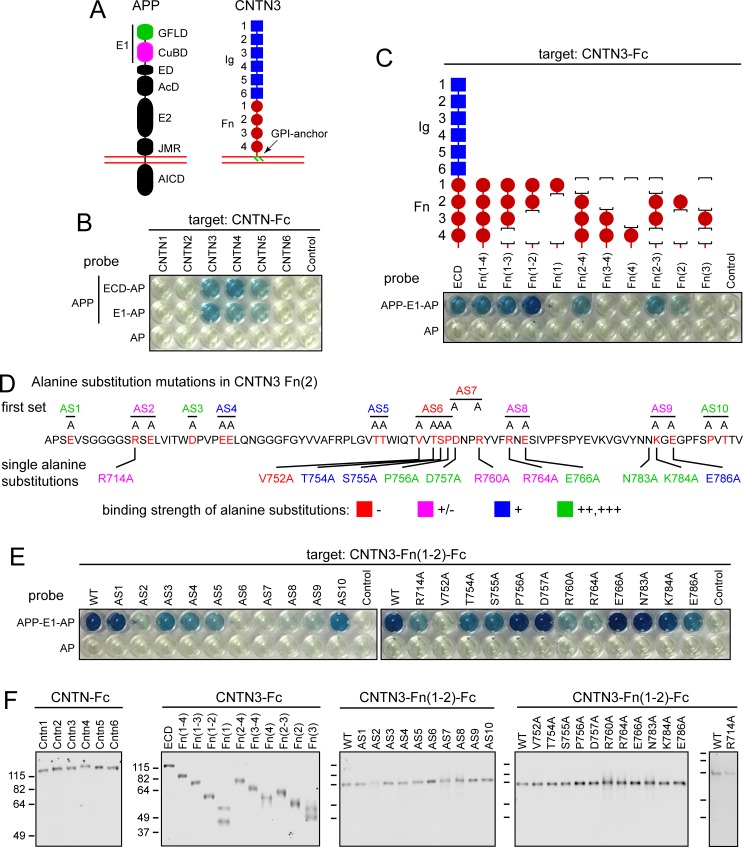
CNTN3 sequences required for binding to APP. (A) Schematic of APP and CNTN3 full-length proteins, showing their domain structures. Red lines indicate the lipid bilayer. Abbreviations for APP: GFLD, growth factor-like domain; CuBD, copper binding domain; ED, extension domain; AcD, acidic domain; E2, central domain; JMR, juxtamembrane region; AICD, APP intracellular domain [2]. Abbreviations for CNTN3: Ig, immunoglobulin domain; Fn, fibronectin domain. (B) Image of a Protein-G coated 96-well tray showing the AP reaction product (blue). The indicated Fc-fusion proteins (“target”) were immobilized by Protein-G binding and then incubated with the indicated APP-AP fusion proteins (the full ECD or the E1 domain) or with AP alone (“probe”). For each CNTN protein the full ECD (including all Ig and Fn domains) was fused to Fc. The concentrations of the different Fc-fusions were adjusted to provide equivalent amounts, as determined by immunoblotting (S1 Fig). The control wells in these and other AP assays were coated with an irrelevant Fc fusion protein. (C) Domain deletions in the ECD of CNTN3-Fc show that Fn(1–2) contains the principal sites of APP-E1-AP binding. The parental fusion protein (WT) has the full CNTN3 ECD fused to Fc. The first Ig domain is adjacent to the signal peptide. (D) Sequence of CNTN3 Fn(2) showing the locations of alanine substitution mutations targeting groups of mostly polar surface amino acids (“first set”; upper sequence) or single amino acids (lower sequence). AS, alanine substitution. (E) 96-well binding assay showing APP-E1-AP binding to the CNTN3 Fn(2) alanine substitution mutations shown in (D) constructed in the context of CNTN3-Fn(1–2)-Fc. (F) Immunoblots of serum-free conditioned medium containing the indicated WT and mutant CNTN-Fc proteins, visualized with anti-human IgG. Mass in kDa is listed for the protein size standards. These are the same for the right-most four blots. Quantification of AP binding is shown in S1A and S1B Fig and is summarized in S1 Table.

Amyloid Precursor Protein (APP) is an evolutionarily conserved vertebrate protein that is expressed in the developing and adult nervous system, as well as in many non-neural tissues [1]. APP has a large extracellular domain (ECD), a single trans-membrane region, and a small cytoplasmic tail. The ECD of the major APP isoform (APP695) consists of the following domains (starting from the N-terminus): (i) the E1 domain, which consists of a growth factor-like domain (GFLD) and a copper-binding domain (CuBD), (ii) the extension domain (ED); (iii) the acidic domain (AcD); (iv) a central domain (E2); (v) the juxtamembrane region (JMR); (vi) the transmembrane domain; and (vii) the intracellular domain (AICD) (Fig 1A) [2]. Over the past 25 years, APP has been the object of intense interest because proteolytic cleavage in the juxtamembrane and transmembrane domains generates the beta-amyloid peptide that accumulates in patients with Alzheimer disease (AD) [3].

The role(s) that APP plays in normal development and physiology are numerous and complex, and their molecular mechanisms are still largely unknown [1]. Gene knockout studies in mice have demonstrated substantially redundant roles for APP and its close homologues, APLP1 and APLP2 (APP-like proteins-1 and -2), in synaptic development, function, and plasticity [4,5], and in dendritic growth, branching, and spine maturation [6–9]. APP has been proposed to function as a cell-surface receptor [10], as a soluble ligand following proteolytic release of its ECD [11,12], and as the source of an intracellular signal via proteolytic release of its C-terminal tail [13,14].

Contactins (CNTNs) are glycosylphosphatidylinositol (GPI) anchored cell-surface proteins with a canonical structure of six amino-terminal Ig domains followed by four fibronectin III-like (Fn) domains (Fig 1A) [15]. There are six CNTN genes in mammals and, for some members of the CNTN family, additional sequence diversity is generated by differential splicing. CNTNs are highly enriched in the nervous system and they play important roles in axon guidance and axon-glial interactions, synapse formation and plasticity, and the organization of multi-protein complexes at the nodes of Ranvier [16,17]. Copy number variation encompassing CNTN genes and sequence variation in or near CNTN genes have been associated with a variety of neuro-developmental disorders, including autism, schizophrenia, Tourette syndrome, and mental retardation [18–21].

A direct biochemical connection between APP and the Contactin (CNTN) family was discovered by Osterfield et al. [22], who showed (i) that among the six CNTNs, APP binds most strongly to CNTN3 and CNTN4, and (ii) that this binding interaction occurs between the N-terminal E1 domain of APP and the four membrane-proximal Fn domains of CNTN. More recently, a functional connection between APP and CNTN4 has been found in a study of retinal ganglion cell axon guidance in mice, in which (i) targeted mutation of *App* or *Cntn4* produced a failure of axonal projections to the nucleus of the optic tract and (ii) *Cntn4* over-expression produced a bias in axonal projections toward the nucleus of the optic tract that required the presence of APP [23].

These studies suggest that a more precise biochemical characterization of the APP-CNTN interaction would be of interest. In the present study, we have used site-directed mutagenesis to define the interfaces on APP and CNTN that mediate their mutual recognition.

## Results

**Fig 2:**
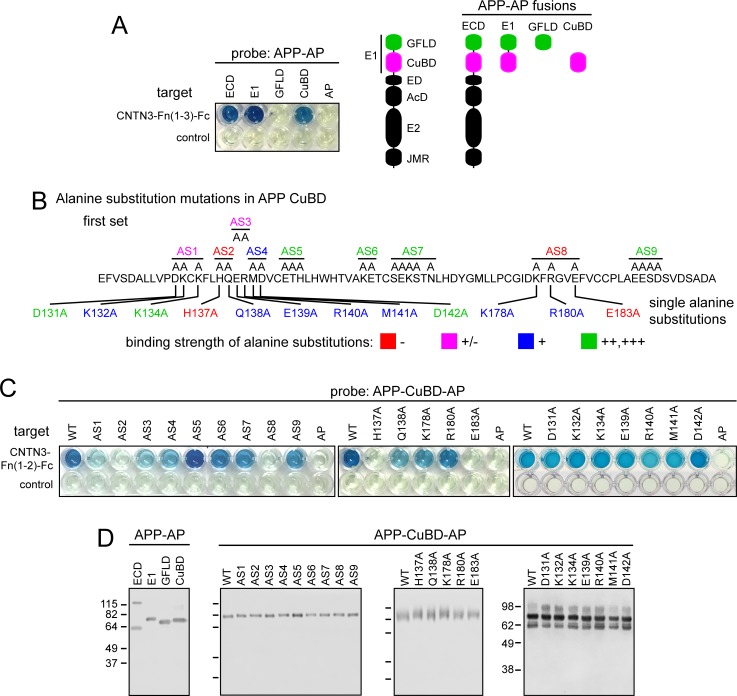
APP sequences required for binding to CNTN3. (A) Left, 96-well binding assay using the AP fusions with the full APP ECD or its amino-terminal domains as probe and CNTN3-Fn(1–3)-Fc as target. Right, schematic of APP ECD domain structure, showing APP-AP fusions. (B) Sequence of APP CuBD showing the locations of alanine substitution mutations targeting groups of mostly polar surface amino acids (“first set”; upper sequence) or single amino acids (lower sequence). (C) 96-well binding assay showing alanine substitution mutations in the CuBD of APP-CuBD-AP binding to CNTN3-Fn(1–2)-Fc. (D) Immunoblots of serum-free conditioned medium containing the indicated WT and mutant APP-AP proteins, visualized with anti-myc mAb. All AP fusion proteins contain a myc-tag between the APP segment and AP. Mass in kDa is listed for the protein size standards, which are the same for the three left-most blots. Quantification of AP binding is shown in S1C–S1E Fig, and is summarized in S1 Table. The concentrations of the different probes were adjusted to provide equivalent amounts of AP enzyme activity, as determined by colorimetric assay.

For in vitro binding assays, fusion proteins consisting of Contactin ECDs or their derivatives joined to the Fc domain of human IgG (“targets”) were immobilized in Protein-G coated microwells, and the microwells were then incubated with soluble fusion proteins consisting of the APP ECD or its derivatives joined to the catalytic domain of human placental alkaline phosphatase (AP) (“probes”). Bound probe was measured with a colorimetric substrate. Targets and probes were produced by transient transfection of HEK293T cells and collected as secreted proteins in serum-free conditioned medium (SFCM). The relative concentrations of targets and probes were estimated by (i) Coomassie Blue staining of protein-G-purified targets following SDS-PAGE and (ii) immunoblotting of SFCM using anti-human IgG (for targets) or anti-myc (for probes, which carry a myc-epitope tag). Examples of these immunoblots are shown in Figs 1F and 2D. To compare signals across multiple targets or probes, the volume of SFCM for each protein was adjusted with serum-free medium to equalize the target or probe concentrations across the set of samples. We note that both AP and Fc are homodimers [24], and therefore target-probe binding should exhibit enhanced avidity compared to binding between the corresponding monomeric target-probe pairs. This effect likely contributes to the high signal-to-noise ratio in the binding assays.

### Identifying amino acids in CNTN3 that are required for APP binding

In an initial experiment, we compared the binding of the full APP ECD and the APP E1 domain, expressed as AP fusions, to each of the six CNTN ECDs, without their GPI anchor motifs, expressed as Fc fusions (Fig 1B). In general agreement with Osterfield et al. [22], both APP probes bound with similar efficiencies, and the relative affinities for the different CNTNs was: CNTN3 ≃ CNTN4 > CNTN5 >> CNTN1, CNTN2, and CNTN6. The one discrepancy with Osterfeld et al. [22] is with CNTN5: Fig 1B shows weak binding whereas Osterfeld et al. [22] saw no binding. Based on this experiment, we focused on CNTN3 for in-depth structure-function studies and we used the APP-E1-AP probe to assess binding to the CNTN3 derivatives.

Deletion of the six N-terminal Ig domains of CNTN3 had no effect on APP-E1-AP binding, thereby localizing the binding interface to the four Fn domains, in agreement with Osterfield et al. [22] (Fig 1C). Deletion of different combinations of Fn domains (in the absence of the Ig domains) revealed an essential role for Fn(2), with Fn(1) providing a several-fold enhancement of the binding signal (Fig 1C). Fn(3) and Fn(4) were found to be dispensable for binding. The signal intensities in this and subsequent binding assays are summarized in the figures and in S1 Table, where -, +/-, +, ++, +++, and ++++ represent the range of intensities from undetectable (-) to maximal (++++). Based on this analysis, alanine substitution mutagenesis experiments were conducted with a target Fc fusion protein containing only the first two Fn domains of CNTN3 (CNTN3-Fn(1–2)-Fc).

To define the amino acids within CNTN3 Fn(2) that mediate APP binding, we designed two rounds of alanine substitution mutagenesis guided by the crystal structure of Fn(2) PDB ID 5I99 [25]. In the first round of mutagenesis, 22 clusters of 1–4 amino acids each were substituted with alanine to narrow down the region(s) of interest. These amino acids were chosen based on their surface location in the Fn(2) crystal structure. Among the 22 mutant Fn(1–2)-Fc proteins, ten were expressed with yields roughly comparable to wild type (WT) Fn(1–2)-Fc (Fig 1F). These ten proteins are assumed to be correctly folded, and they are labeled alanine substitution (AS)1-10 in Fig 1D, upper sequences. AS2, AS6, AS7, AS8, and AS9 exhibited greatly reduced binding to APP-E1-AP, suggesting a role for one or more amino acid side chains in these clusters (Fig 1E, left panel and S1A Fig).

In the second round of mutagenesis, each of the 13 amino acids that were mutated in CNTN3-Fn(1–2)-Fc AS2, AS6, AS7, AS8, and AS9 was individually mutated to alanine (Fig 1D, lower sequences). Twelve of the 13 individual alanine substitution mutants were secreted with yields roughly comparable to WT (Fig 1F). E716A was produced with a poor yield and was not further studied. Among the 12 single alanine mutants, V752A showed undetectable binding, and R714A, R760A, and R764A showed greatly reduced binding (Fig 1E, right panel and S1B Fig), indicating an important role for each of these four amino acids in APP binding. It is interesting that three of these four amino acids are arginine.

### Identifying amino acids in APP that are required for CNTN3 binding

We applied a similar strategy to localize the CNTN3 binding site within APP E1. We first tested the binding properties of the two subdomains of E1 –GFLD and CuBD–and observed that CuBD alone conferred binding to CNTN3 with essentially no contribution from GFLD (Fig 2A). APP CuBD contains a Type 2 non-blue copper center in which His147, His151, and Tyr168 bind the central copper ion [26].

To further localize the amino acids within APP CuBD responsible for CNTN3 binding, nine clusters of 1–5 alanine substitution mutations were constructed in nearby surface residues in the APP-CuBD-AP fusion protein, based on the CuBD and E1 crystal structures PDB ID 2FKL and PDB ID 3KTN [26, 27] (Fig 2B AS 1–9, upper sequences). All nine variants were secreted with yields roughly comparable to WT (Fig 2D). In the binding assays with WT CNTN3-Fn(1–2)-Fc, APP-CuBD-AP mutants AS2 and AS8 showed no binding, AS1 and AS3 showed strongly reduced binding, and AS4 showed modestly reduced binding (Fig 2C, left panel and S1C Fig). Interestingly, mutating His147 (in AS5), which is one of the copper-liganding residues, did not affect binding.

In the second round of mutagenesis, each of the 12 amino acids that were mutated in APP-CuBD-AP AS1, AS2, AS3, AS4, and AS8 was individually mutated to alanine (Fig 2B, lower sequences). All 12 mutants were expressed with yields roughly comparable to WT (Fig 2D), and, among these, H137A and E183A showed undetectable binding, and K132A, Q138A, E139A, R140A, M141A, K178A, and R180A showed a several fold reduction in binding (Fig 2C, right two panels and S1D and S1E Fig).

### Spatial locations of amino acids that are essential for APP-CNTN3 binding

**Fig 3:**
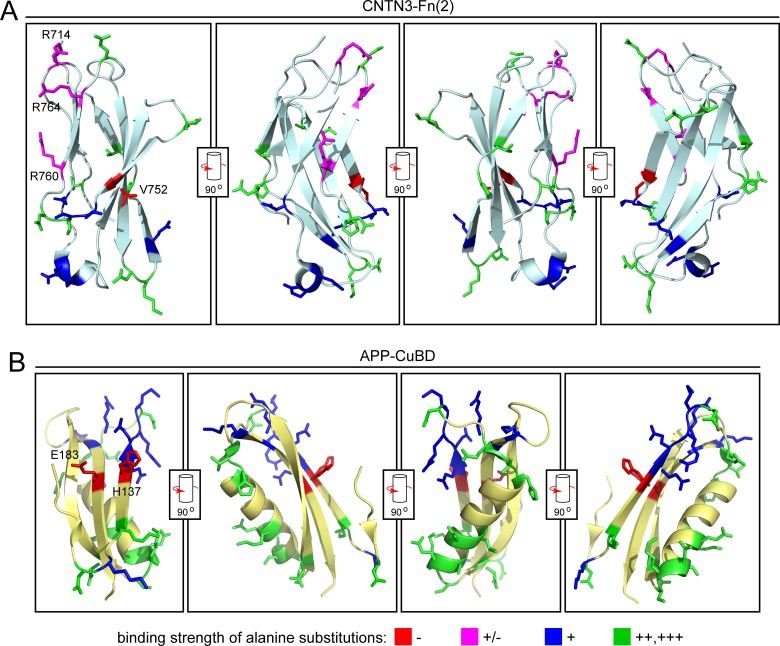
Amino acids that are critical for APP-CNTN3 binding displayed on the 3-dimensional structures of CNTN3-Fn(2) and APP-CuBD. (A) Locations of alanine substitutions in CNTN3-Fn(2) PDB ID 5I99 [25], color coded by their effect on APP-E1-AP binding. (B) Locations of alanine substitutions in APP CuBD PDB ID 2FKL [26], color coded by their effect on CNTN-Fn(1–2)-Fc binding. The cylinders and arrows show the 90 degree rotations that relate the images in adjacent panels. Only data from CNTN-Fn(1–2)-Fc or APP-CuBD-AP fusion proteins that were secreted well are shown. In the leftmost panels, side chains are labeled for those amino acid that are most critical for binding, i.e., those for which alanine substitution severely impairs binding (categories: +/- (magenta) and–(red)).

The locations of the alanine substitution mutations were mapped onto ribbon diagrams of CNTN3 Fn(2) and APP CuBD, color-coded by their effect on binding (Fig 3). The four CNTN3 Fn(2) substitutions that most severely disrupt binding reside on one face of the Fn(2) domain (Fig 3A). Similarly, the two APP CuBD substitutions that most severely disrupt binding reside on adjacent strands of a beta-sheet on one face of the CuBD (Fig 3B). Five of the six APP CuBD substitutions with several-fold lower binding efficiency (Fig 3B, color-coded blue) reside adjacent to these two critical positions, and the sixth resides further away on the same face of the CuBD. As the mutations with the greatest effect on CNTN3 Fn(2) and APP CuBD binding reside on one face of each of these domains, the most parsimonious explanation for the mutagenesis data is that these two faces interact.

## Discussion

In this study, we have confirmed and extended the findings of Osterfeld et al. [22], by showing that: (i) binding of the full ECD or the E1 domain of APP occurs with affinities CNTN3 ≃ CNTN4 > CNTN5 >> CNTN1, CNTN2, and CNTN6; (ii) in CNTN3, APP binds to Fn(2); (iii) in APP, CNTN3 binds to the CuBD; and (iv) the most important amino acids for binding reside on one face of CNTN3-Fn(2) and on one face of APP-CuBD. The first of these observations is consistent with the relative sequence similarity among members of the Contactin family: CNTN1-2 and CNTN3-6 form two main groups of related sequences, and, within the second group, CNTN3 and CNTN4 are the most closely related pair [15].

Multiple interacting protein partners have been identified for both the APP/APLP and CNTN families, and it seems likely that both protein families function via a network of cis- and trans-acting partners. Additionally, both families generate cell surface and secreted protein isoforms, implying that they can act both locally or at longer range. As noted in the Introduction, only one study has explored functional interactions between mammalian CNTN and APP in vivo [23]. In addition to the retinal ganglion cell axon guidance defects found in *Cntn4* knockout mice [23], glomeruli in the olfactory exhibit reduced specificity of innervation by olfactory receptor neurons [28]. By analogy with the experiments of Osterhout et al. [23], it would be of interest to determine whether *App* or *Aplp* deletion or over-expression also affects the olfactory bulb phenotype. A similar approach could also be used to look for *App* or *Aplp* affects on the phenotypes associated with targeted mutation in *Cntn5*, which include defects in the auditory system [29], the development of axo-axonic synapses in the spinal cord [30], and dendritic patterning among retinal ganglion cells [31].

Invertebrate genomes also code for APP and CNTN family members. Conveniently, the two best studied invertebrate model organisms, *C*. *elegans* and *D*. *melanogaster*, each have only a single APP homologue (APL-1 in *C*. *elegans* [32] and APPL in *D*. *melanogaster* [33]) and a single CNTN homologue (RIG-6 in *C*. *elegans* [34] and CONT in *D*. *melanogaster* [35]). At present, there are no reports of APP-CNTN interactions in *C*. *elegans* or *D*. *melanogaster*. However, in the developing nervous system of the hawkmoth *Manduca sexta*, interactions between APPL (the *M*. *sexta* homologue of APP), which is expressed by migratory neurons, and MsContactin (the *M*. *sexta* Contactin homologue), which is expressed on adjacent glia, are important for guiding neuronal migration and outgrowth [36]. The *M*. *sexta* experiments suggest that there may be an ancient evolutionary connection between these two families of proteins. It would be of interest to determine whether the *M*. *sexta* APPL and MsContactin proteins exhibit the same mode of binding as the mammalian APP and CNTN3 proteins.

The multitude of protein partners for both APP/APLP and CNTN family members complicate the interpretation of genetic interaction experiments based on gene deletion or over-expression because such gross genetic perturbations can produce indirect effects that are mediated by protein partners other than the ones under consideration. The present experiments lay the groundwork for a more incisive analysis of CNTN-APP interactions in vivo by defining a minimal set of mutations that alter their mutual binding without affecting protein structure or stability. Thus, introducing a minimal set of mutations into either of these two genes by CRISPR/Cas9 editing should produce an allele in which the APP-CNTN interaction is selectively eliminated, while preserving most or all of the binding interactions with other partners.

## Materials and methods

### Constructs

Fc and AP fusions were cloned into the pRK5 vector for expression in HEK/293T cells [37]. For the Fc-fusion proteins, the open reading frame consisted of (i) the native signal peptide or the mouse Frizzled8 signal peptide, (ii) the full ECD or the subdomain of interest, (iii) a 22 amino acid linker corresponding to the region immediately distal to the cysteine-rich region of mouse Frizzled8, and (iv) the Fc region of human IgG1 starting from the hinge [38]. For the AP-fusion proteins, the open reading frame consisted of: (i) the native signal peptide or the mouse Frizzled8 signal peptide, (ii) the full ECD or the subdomain of interest, (iii) a glycine/serine linker followed by a myc epitope tag, and (iv) the coding sequence of human placental alkaline phosphatase (PLVAP) lacking the GPI-anchoring sequences [39].

### CNTN and APP inserts

Full-length CNTN2, CNTN4.1, CNTN5, and APP ECDs were amplified from a mixture of prenatal mouse embryo and neonatal mouse brain cDNA by nested PCR. Mouse CNTN1, CNTN3, and CNTN6 ECD-Fc constructs were a generous gift of Dr. Davide Comoletti. CNTN3 and APP domain deletions and alanine substitutions were generated by PCR using the cloned ECD sequences as templates.

### Cell culture and transfection

HEK/293T cells (ATCC CRL-11268) were transiently transfected with polyethylenimine (PEI). For small-scale transfections, HEK293T cells were grown in 12-well plates. 24 hours after transfection, wells were washed twice with 1 ml serum-free medium (SFM), and then cultured for an additional 24 hours in 0.5 ml SFM. The serum-free conditioned medium (SFCM) was centrifuged at 5000xg for 5 minutes, and 20 ul of the supernatant was used for immunoblotting. Plasmids that produced ~1 ug/ml protein in the SFCM were used for large-scale transfection. For large-scale transfections, HEK/293T cells were plated in 10 cm dishes and processed as described above, except that (i) washes were performed three times with 5 mls SFM, (ii) 5 mls SFCM was generated per plate, and (iii) SFCM production was continued with a fresh 5 ml of SFM for an additional day, if more protein was needed. SFCM was filtered through a 0.2 um filter, and stored at 4°C with sodium azide added to 0.01%.

### Binding assays

For estimating Fc fusion protein concentrations, 1 ml of SFCM was captured on Protein-G sepharose beads (Thermo-Fisher), and analyzed by SDS-PAGE and Coomassie Blue staining. Protein concentrations were compared to a bovine serum albumin (BSA) dilution series. Relative protein concentrations for WT and mutant Fc- and AP-fusion proteins were determined using the LI-COR fluorescent immunoblotting system (LI-COR Biosciences), and the volumes of SFCM adjusted accordingly to equalize the concentrations across WT and mutant versions in the binding assays. For binding assay, the wells of a Protein-G coated 96-well plate (Thermo-Fisher Scientific/Pierce) were washed twice with PBS, 100 μl of volume-adjusted conditioned medium containing each Fc-fusion proteins was added per well, and then the plate was rotated horizontally for 1.5–2 hours at room temperature. The medium was removed and the wells washed 5 times in PBS with 0.5% Triton X-100, 0.1% bovine serum albumin (PBSTA). 100 μl of volume-adjusted conditioned medium containing the AP-fusion protein was added per well, and the plate was rotated for 1.5–2 hours at room temperature, and then washed 5 times with PBSTA. Five minutes before use, a 1:1 mixture of the BluePhos Microwell Substrate (Kirkegaard and Perry Laboratories) was prepared and 100 μl was added to each well. Absorbance at 620 nm was recorded at 5 minutes intervals at room temperature using a SpectraMax M3 Microplate Reader (Molecular Devices) for 60–90 minutes. Plates were photographed after the incubation was complete.

## Supporting information

S1 Fig Quantification of the binding reactions between APP-E1-AP and alanine substitution mutants of CNTN3-Fn(1–2)-Fc (A and B) and between alanine substitution mutants of APP-CuBD-AP and CNTN3-Fn(1–2)-Fc (C-E). The AP reaction utilized the Blu-Phos Microwell substrate and was monitored at 5-minute intervals over 60–90 minutes at room temperature with a 96-well plate reader.
(EPS)

S1 Table Binding properties of CNTN3 and APP. The CNTN3 binding data refer to Fig 1 and S1A and S1B Fig. The APP binding data refer to Fig 2 and S1C–S1E Fig.
(DOCX)
