# Supplementary material for: Defining the binding interface of Amyloid Precursor Protein (APP) and Contactin3 (CNTN3) by site-directed mutagenesis
Source: PLoS One. 2019 Jul 18;14(7):e0219384. doi: 10.1371/journal.pone.0219384 (PMC6638891; doi:10.1371/journal.pone.0219384)
Supplement: S1 Table — The CNTN3 binding data refer to Fig 1 and S1A and S1B Fig. The APP binding data refer to Fig 2 and S1C–S1E Fig. (DOCX) [file pone.0219384.s002.docx]

CNTN3 domain deletions

pXP Domains retained Level of binding to APP-E1

53 full ECD +++

79 Fn(1-4) +++

94 Fn(1-3) +++

95 Fn(1-2) ++++

96 Fn(1) -

97 Fn(2-4) ++

98 Fn(3-4) -

99 Fn(4) -

100 Fn(2-3) ++

101 Fn(2) +

102 Fn(3) -

CNTN3 first set of alanine substitutions

pXP Mutation Level of binding to APP-E1

95 WT +++

135 AS1 +++

136 AS2 +/-

137 AS3 ++

138 AS4 +

140 AS5 +

122 AS6 -

141 AS7 -

142 AS8 +/-

144 AS9 +/-

127 AS10 ++

CNTN3 single alanine substitutions

pXP Mutation Level of binding to APP-E1

95 WT ++

1521 R714A +/-

1522 E716A not expressed

169 V752A -

170 T754A +

171 S755A +

172 P756A +++

173 D757A +++

174 R760A +/-

175 R764A +/-

176 E766A ++++

177 N783A +++

178 K784A ++

179 E786A +

APP domain deletions

pXP Domains retained Level of binding to CNTN3-Fn(1-2)

54 full ECD ++

82 E1 +++

107 GFLD -

108 CuBD ++

APP-CuBD first set of alanine substitution

pXP Mutation Level of binding to CNTN3-Fn(1-2)

108 WT ++

128 AS1 +/-

145 AS2 -

146 AS3 +/-

147 AS4 +

130 AS5 +++

131 AS6 ++

132 AS7 ++

133 AS8 -

134 AS9 ++

APP-CuBD single alanine substitutions

pXP Mutation Level of binding to CNTN3-Fn(1-2)

108 WT +++

715 D131A +++

716 K132A +

717 K134A +++

718 E139A +

719 R140A +

720 M141A +

721 D142A +++

165 H137A -

166 Q138A ++

148 K178A ++

149 R180A +++

150 E183A -
